# Vascular Fluorescence Imaging Control for Complex Renal Artery Aneurysm Repair Using Laparoscopic Nephrectomy and Autotransplantation

**DOI:** 10.1155/2014/563408

**Published:** 2014-08-10

**Authors:** Matteo Tozzi, Luigi Boni, Gabriele Soldini, Marco Franchin, Gabriele Piffaretti

**Affiliations:** ^1^Vascular Surgery, Department of Surgery and Center for Research on Organ Transplantation, Insubria University School of Medicine, Circolo University Hospital, 21100 Varese, Italy; ^2^General Surgery 1, Department of Surgery and Center for Research on Organ Transplantation, Insubria University School of Medicine, Circolo University Hospital, 21100 Varese, Italy; ^3^Vascular Surgery, Department of Surgery and Morphological Sciences, Insubria University School of Medicine, Circolo University Hospital, Via Guicciardini 9, 21100 Varese, Italy

## Abstract

Intraoperative fluorescent imaging using indocyanine green enables vascular surgeons to confirm the location and states of the reconstructed vessels during surgery. Complex renal artery aneurysm repair involving second order branch vessels has been performed with different techniques. We present a case of ex vivo repair and autotransplantation combining the advantages of minimally invasive surgery and indocyanine green enhanced fluorescence imaging to facilitate vascular anatomy recognition and visualization of organ reperfusion.

## 1. Introduction

Operative repair of renal artery aneurysm (RAA) may be accomplished by different techniques; actually, endovascular stent-grafting or embolization procedures are attractive alternative for the RAAs treatment [1, 2]. However, RAA beyond the renal artery bifurcation may require an open treatment with in vivo or ex vivo surgical repair [1, 3]. In case of ex vivo repair, laparoscopic donor nephrectomy has currently become widely accepted because of the minimally invasiveness and lower morbidity rate if compared to the conventional approach for living renal donation [4, 5].

Intraoperative vascular quality control during renal transplantation is not performed on a routine basis, while duplex ultrasound evaluation of the organ perfusion is generally performed postoperatively [6]. A technical advancement has been recently introduced in general surgery, that is, the intraoperative use of fluorescent imaging using indocyanine green (ICG); this is an intriguing technique for easier intraoperative recognition of vascular anatomy and for evaluating organ perfusion that has been reported also in kidney transplantation [6–9].

We report a case of a complex RAA treatment using laparoscopic nephrectomy, ex vivo aneurysmectomy, and autotransplantation with the intraoperative use of ICG fluorescent imaging to optimize the visualization of vascular anatomy and control the revascularization after vascular repair.

## 2. Case Report

**Figure 1:**
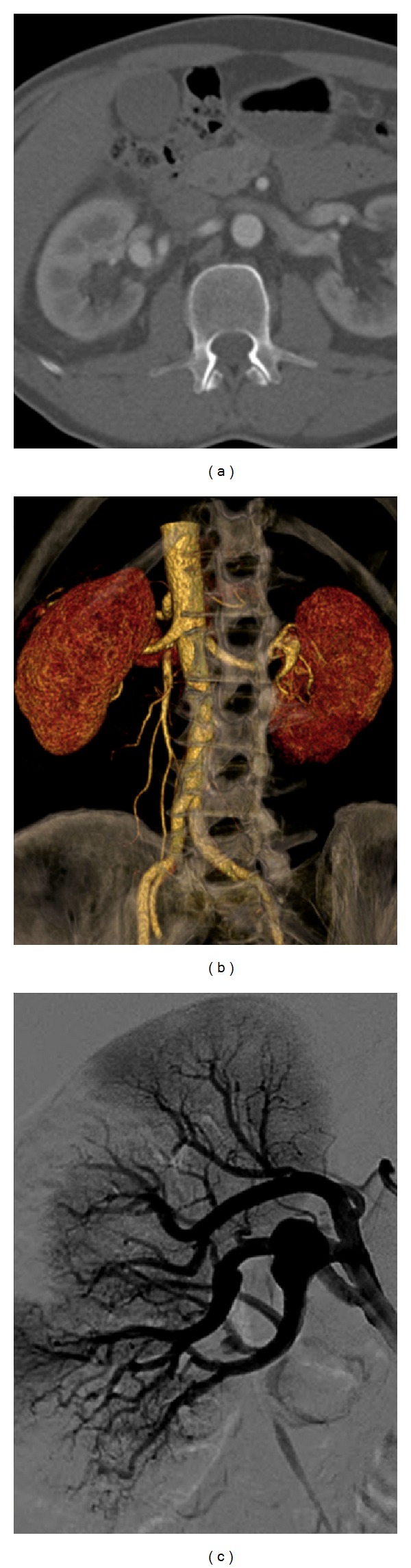
Preoperative 3D volume rendering computed tomography scan (a), with volume rendering 3D reconstruction (b) and angiography (c) showing the 20 mm fusiform aneurysm (*arrows, *a-b-c) of the secondary inferior branch of the right renal artery.

A 53-year-old man with a history of recurrent right flank pain was referred to our department following the diagnosis of a right renal artery aneurysm. Medical history was relevant for psoriatic arthritis. Preoperative computed tomography-angiography (CT-A) showed a 20 mm juxta-hilar RAA, type 5, according to the classification of Henke et al. [10] (Figures 1(a)–1(c)). Baseline serum creatinine level was 0.8 mg/dL. A first attempt to treat the RAA with an endovascular stenting using a flow-modulator stent failed. Postoperative CT-A identified the occlusion of another branch artery, while the renal sequential scintigraphy showed two iatrogenic parenchymal ischemic areas with normal perfusion/excretion function. Hence, a decision was made to proceed with an ex vivo repair with an autotransplantation.

### 2.1. Surgical Technique

**Figure 2:**
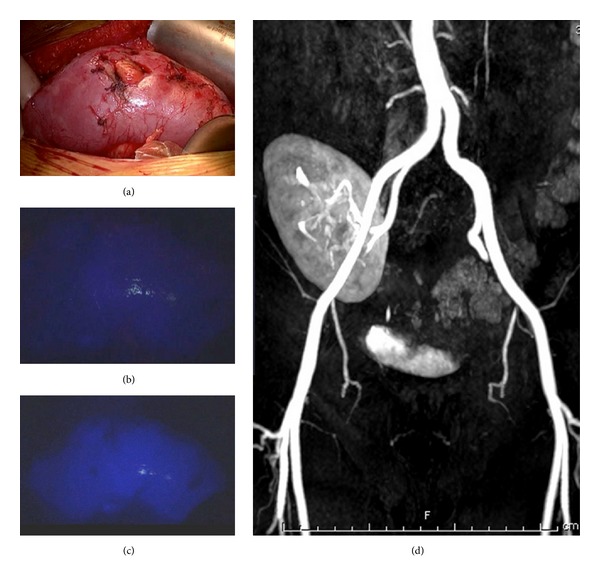
Intraoperative aspect of the transplanted kidney (a) and visualization of the parenchyma at the time of indocyanine green injection (b) and after the revascularization with real-time fluorescence imaging (c). Follow-up control with magnetic resonance angiography (d).

Right nephrectomy was performed as previously described [4, 5]; it was completed using a “hockey stick” laparotomy, the same that was subsequently used for autotransplantation. Total time of warm ischemia was 40 seconds. During bench back surgery, the kidney was flushed with hypothermic University of Wisconsin solution; the aneurysm was excised and vascular reconstruction completed with a primary anastomosis with no intervening vein or other grafts. At this time, we identified the thrombosis of a medial branch artery due to a narrowing dissecting flap caused during the endovascular procedure. At the completion of the vascular anastomosis to the external iliac vessels, 5 mL of ICG at a concentration of 0.3 mg/mL/kg was injected intravenously. For ICG fluorescent imaging, we used a laparoscopic system (TELECAM SL II - Storz; Tuttlingen-GER), a 10 mm laparoscope applicable for white light (WL), autofluorescence imaging, and ICG-imaging. Fluorescent imaging confirmed the homogeneous vascular perfusion of the kidney. The ischemic lesions of the lower pole were no longer observed at the completion fluorescent imaging (Figures 2(a)–2(c)). Autotransplantation with a Lich-Gregoir ureteroneocystostomy was then completed in standard fashion [10]. Overall, duration of intervention was 430 minutes and cold ischemia time was 105 minutes. Histological evaluation of the renal aneurysm revealed marked atherosclerotic changes.

### 2.2. Postoperative Outcome and Follow-Up

No complication occurred. He was ambulatory on postoperative day 3 and then was discharged on postoperative day 10. He was last seen 6 months after the procedure alive and well, asymptomatic with serum creatinine of 0.76 mg/dL; contrast-enhanced magnetic resonance imaging confirmed the successful vascular repair, also confirmed by the fact that the preoperative parenchymal ischemic areas disappeared (Figure 2(d)).

## 3. Discussion

Current indications for the treatment of RAAs should take into account several factors which are patient and aneurysm related [1–3, 10]. In our case, we had two reasons to recommend RAA repair: a diameter of >20 mm, remarkable if we consider the involvement of a branch artery, and flank pain presumed to be related to the aneurysm [2, 3, 10].

The results of endovascular management of renal artery occlusive disease comparable to those of open repair have led to consider RAAs an attractive target for endovascular therapy [3, 11]. Nevertheless, multiple determinants should influence the decision making process: the aneurysm position and the presence of important branches arising from or in close proximity to the aneurysm are of utmost importance [11–13]. In those circumstances, major concerns remain about the broad applicability of stent-grafting or flow-modulator stent because every attempt should be made to preserve as much of the kidney as possible [10–13]. We also had to deal with the young age in our patient, since the long-term outcomes of endovascular therapies in renal vessels remain uncertain [2, 11]. Having weighed all these factors and following the failed endovascular attempt, open surgery remained our ultimate option.

Anticipated unreconstructable renal artery, ruptures, intraparenchymal location, or irreparable renal ischemia has been treated also with nephrectomy [1–3, 10–12]. This was not an option in our mind because of the young age of the patient, the absence of aneurysm-related hypertension, and last but not the least the satisfactory results of reconstructive surgery in that aneurysm location. Henke et al. [10] reported no major differences in long-term patency or need for secondary interventions between patients who had aneurysmectomy and bypass versus patch closure.

Owing to the complicated renal vascular anatomy operative strategy should be tailored according to the type of lesion and experience of the operator [2–15]. An ex situ repair has been preferred for a complex operation site, such as branch aneurysm with the need of repair at or within the renal hilum [9, 15–19]. This type of operation is generally accomplished with the dissection of the vascular pedicle, while the ureter is mobilized but left intact [13, 14]. We believe this technique is demanding, is associated with significant incisional morbidity, and is time consuming with longer cold ischemia than bench surgery [12, 14]. Further, we believe the best results are obtained with a technique with which surgeon is confident; therefore, we took advantage of the experience we gained in kidney transplantations. Laparoscopic nephrectomy limited the invasiveness of the intervention, and kidney perfusion was optimized with the best available solution, while vascular reconstruction was completed with ease without grafting during the back bench surgery [17, 18].

Complex RAA repair combining laparoscopic nephrectomy and autotransplantation is not new: Gallagher et al. [14] already published a successful case series of seven patients. An interesting aspect added in our case is the use of the fluorescent angiography which has been already adopted during kidney transplantation [6–10]. In our case, major advantages were a reliable real-time roadmap of the renal vessels and ureter anatomy and intraoperative quality control of the vascular reconstruction and kidney perfusion. Further, it is safe because radiations are not requested and the risk of adverse reactions to the ICG injection is very low.

Graft perfusion is of utmost importance since the functions of a transplanted kidney are critically dependent on the quality of blood supply. Although duplex ultrasound may reveal areas of hypoperfusion undetected intraoperatively, currently the assessment of organ reperfusion is left to the visual judgment of the operating surgeon [6–9, 19]. Fluorescent angiography produces a more sensitive real-time assessment of the organ perfusion and may detect other problem areas in the graft [6–9]. Hoffmann et al. [7] emphasized the important role played by ICG fluorescent imaging in providing useful information for intraoperative decisions: ICG dictated a graft repositioning for a large perfusion defect that was unremarkable to visual inspection. This observation finds support in our case: in fact, the perioperative ischemic areas detected in the preoperative scintigraphy were determined by a stenosis of a medial branch artery. The correction of this stenosis was effective and decisive as confirmed by the disappearance of these areas at the completion fluorescent angiography.

There are some limitations with this technique: first and foremost is the limited ability of near-infrared light to penetrate tissues; hence, it is not useful for percutaneous procedure. Second is the absence of well-defined thresholds of the fluorescent signals: the concentration or amount of ICG solution, time and speed of bolus injection, and patient parameters such as body weight all interact with the imaging effect and quality [20]. Apart from these shortcomings, in our case, these disadvantages were partially lessened with the capacity of laparoscopy to be close to the tissues. Nonetheless, it is less invasive, easier to use, and less risky than ionizing radiation using contrast medium to identify the pattern of the vasculature architecture at the end of the vascular anastomosis [6–9]. With regard to the increasing use of marginal grafts that crucially require optimal perfusion conditions, this technique might be useful in the next future to enrich renal transplantation surgery [7].

## 4. Conclusion

Complex RAA repair using laparoscopic nephrectomy with ex vivo repair and autotransplantation has been shown to be feasible and safe. Indocyanine green mediated fluorescent imaging seems a potentially helpful adjunct for the evaluation of graft reperfusion after transplantation: the application of this technology emphasized the kidney perfusion and confirmed the absence of ischemic areas within the graft.
